# Best practice for forensic anthropology reporting: a statement by the Board of the Forensic Anthropology Society of Europe (FASE)

**DOI:** 10.1007/s00414-026-03903-0

**Published:** 2026-07-08

**Authors:** Eugénia Cunha, Anja Petaros, Desiré Brits, Cristina Cattaneo, Elena Kranioti, Ann H. Ross, Pascal Adalian, Zuzana Obertova, Hans H. de Boer

**Affiliations:** 1 National Institute of Legal Medicine and Forensic Sciences, Lisbon, Portugal; 2 University of Coimbra, Centre for Functional Ecology, Laboratory of Forensic Anthropology, Department of Life Sciences, Coimbra, Portugal; 3 Division of Forensic Medicine Linköping, Department of Forensic Medicine, Swedish National Board of Forensic Medicine, Linköping, Sweden; 4 Division of Clinical Chemistry and Pharmacology, Department of Biomedical and Clinical Sciences, Faculty of Medicine, Linköping University, Linköping, Sweden; 5 Human Variation and Identification Research Unit (HVIRU), Department of Anatomical Sciences, School of Biomedical Sciences, Faculty of Health Sciences, University of the Witwatersrand, Johannesburg, South Africa; 6 LABANOF, Laboratory of Forensic Anthropology and Odontology, Section of Forensic Medicine, Department of Biomedical Sciences for Health, University of Milan, Milan, Italy; 7 Forensic Medicine Unit, Department of Forensic Sciences, University of Crete, Heraklion, Greece; 8 Department of Biological Sciences, North Carolina State University, Raleigh, NC USA; 9 Aix Marseille Univ, CNRS, EFS, ADES, Marseille, France; 10 Centre for Forensic Anthropology, School of Social Sciences, The University of Western Australia, Crawley, Australia; 11 Victorian Institute of Forensic Medicine, Southbank, VIC Australia; 12 Department of Forensic Medicine, Monash University, Southbank, VIC Australia

**Keywords:** Forensic anthropology, Report writing, Guidelines, Consensus, Forensic practice

## Abstract

The case report is the primary means by which forensic anthropologists communicate the outcomes of their examination. Despite their central role, considerable variability currently exists in the content, structure, and clarity of such reports. Drawing on published guidelines, forensic science standards, and the collective experience of an international group of practitioners from the Board of the Forensic Anthropology Society of Europe (FASE), this paper presents practical recommendations for forensic anthropology reporting. It outlines the fundamental aims and principles of reporting, identifies essential report elements, and distinguishes minimum requirements from discretionary components. Emphasis is placed on scientific integrity, transparency of methods and reasoning, and clear communication to a diverse audience. These recommendations are meant to be applicable across jurisdictions and professional contexts, including independent practice, while remaining aligned with contemporary forensic science standards. By promoting consistency and clarity in reporting, these recommendations aim to support the reliability, interpretability, and admissibility of forensic anthropological evidence, and to strengthen the contribution of forensic anthropology to medico-legal investigations.

## Introduction

The forensic anthropology case report is the principal method by which forensic anthropologists communicate the results of their analyses. It is therefore a central and indispensable component of forensic anthropology practice. It contains essential information to help guide a medico-legal investigation and is often used as evidence in court. Therefore, it should be meticulously prepared, comprehensive, and factually correct.

Writing a good report can be challenging. The document should meet specific administrative and legal requirements, or else it may become inadmissible as forensic evidence. Beyond administrative compliance, a good report demands a delicate balance between comprehensiveness, efficiency, and readability. This can be difficult, since forensic anthropological analyses often involve complex statistical methods, descriptive observations, and case-specific limiting factors. When these aspects are not sufficiently addressed, this can lead to omissions, errors, and misunderstandings. An incomplete or unclear report can ultimately undermine the value of the examination, be potentially challenged by other parties, and detrimentally affect medico-legal investigations and the administration of justice.

To assist practitioners, several publications address reporting specifically for forensic anthropology (e.g., [1–11]). However, many of these focus on high-level principles (e.g., objectivity, impartiality, clarity) without providing practical advice on how to achieve them. In addition, they may be aimed at specific jurisdictions or groups of professionals, for instance those employed within forensic institutes. In addition, since forensic anthropologists with active casework operate within diverse professional contexts [12], such recommendations may not be applicable for practitioners pertaining to other jurisdictions, those who work independently, or those principally employed in academia.

As part of their certification process, The Forensic Anthropology Society of Europe (FASE) has reviewed numerous reports from many countries and jurisdictions and observed considerable variability in reporting quality and content. This underscores the need for clear, practical, and widely applicable reporting recommendations.

The purpose of this paper is to provide recommendations regarding basic principles and elements of a forensic anthropology case report. A distinction is made between minimum requirements and discretionary elements, to acknowledge the inevitable variation in the scope of practice and professional context among practitioners. This is to ensure the applicability of the recommendations across a broad spectrum of medico-legal contexts and case types, while upholding methodological and inferential transparency.

## Materials and methods

The recommendations in this document are based on a review of publications and guidelines on forensic scientific and forensic anthropology reporting, as well as the relevant scientific, regulatory, and professional literature [1–31], combined with the shared experiences of the multi-national author group. Several members of the author group are part of the Certification Working Group of the Forensic Anthropology Society Europe (FASE).

To align forensic anthropology reporting with other types of forensic sciences, these recommendations adopt the terms and definitions suggested in the ISO standard ‘Forensic Sciences’ (ISO 21043-1: Forensic Sciences - vocabulary [13]):

- **Examination**: part of the forensic process consisting of the recovery and analysis of *items*, and the *interpretation* of *observations* obtained from the *analysis* of *items*.
- **Item**: object, substance, material or mark that is collected, derived or sampled as part of the forensic process. In forensic anthropology, this will often be a (part of a) skeletal element. Other examples are radiological or photographic images.
- **Analysis**: part of the *examination* consisting of detecting, measuring or comparing the properties of *items*, in order to obtain *observations*. Analysis can be either instrumental, human perception-based, or a combination of the two.
- **Observation**: result of *analysis* of *items* or the scene. An observation can be the result of human perception-based analysis, instrumental analysis, or a combination of the two.
- **Interpretation**: Part of the *examination* that uses professional judgement, logic, expertise, and relevant data and information and, if applicable, statistical models to infer the meaning of *observations* so as to provide *opinions* with respect to questions asked. As outlined in [21] “Conclusion” is not used for the output of interpretation. Also, neither “result” nor “finding” is used for the output of analysis nor for the output of interpretation.
- **Opinion**: the examiner’s judgement based on the *interpretation* of *observations*.

## Aims and basic principles of reporting

Notwithstanding variations in professional practice and legal context, the principal objective of a forensic anthropology report is always the same: to communicate specialized forensic anthropological knowledge in a clear and accessible manner.

More specifically, each report serves the following purposes:

- documenting the source and the scope of the examination;
- identifying the author(s) of the report;
- listing the examined items;
- describing and justifying the analysis; and
- presenting the observations and any opinions derived from them, within the context of the case.

These should be described in sufficient detail to allow for independent verification and cross-examination.

From these general aims, some principles can be derived that apply to every report.

### Neutrality

Neutrality (or impartiality) is a fundamental principle of all forensic expert reporting. The forensic anthropologist’s primary obligation is to scientific integrity, not to the referring agency or any party involved in the legal proceedings. Any statement or formulation that suggests otherwise can seriously undermine the credibility of the report. To protect neutrality, the observations and interpretations of the examination should remain confidential, at least until the report is finalized.

Many jurisdictions have laws or regulations in place that govern expert witness statements and that stipulate how neutrality and confidentiality should be maintained (e.g., [20, 27, 28]). When applicable, the report should explicitly state that the author has read and agrees to adhere to them.

### Scientific integrity

All interpretations in the report should be underpinned by valid, robust, and transparent scientific analyses and reasoning. The report should have a clear structure, separating contextual information, examined items, analytical methods, observations, and opinions. The distinction between observations and opinions is especially relevant to allow colleagues or other practitioners to form their own independent opinions. A structured approach may also help identify sources of contextual or cognitive bias, as it helps to show the flow of information and inferential process of the forensic anthropologist.

Scientific integrity also requires consideration of limitations that reasonably may affect the validity of the examination. Limiting factors can be encountered in almost any part of the analysis, such as deficiencies in background information, poor preservation status of the remains, unavailable or limited reference data, or uncertainty/error associated with analytical methods. Clearly communicating the presence and implications of these limitations helps the reader to evaluate the reliability of the observations and opinions.

Practitioners should never provide opinions outside their area of expertise. It is, however, acknowledged that some practitioners are also trained in forensic pathology, radiology, or odontology, meaning that boundaries of expertise may differ among experts.

### Clarity

Forensic anthropological reports can be used by a broad audience, including colleagues, other forensic experts, law enforcement, judicial officers, lawyers, and members of the public (including next of kin), all with different levels of anatomical and forensic knowledge. The language in the report should therefore be comprehensible to non-specialists, without sacrificing informative value, and retaining sufficient detail for peer review.

To achieve this, language should be used carefully. Complex and convoluted phrasing should be avoided, but oversimplification may undermine the scientific rigor of the report. Discipline-specific terms should be defined, either in-text or in a glossary. The use of tables, photographs, and diagrams facilitates understanding. Long, complex tables are better included in an appendix than the body of the report.

The use of vague, ambiguous, or suggestive language should be avoided. In addition, emotive, inflammatory, or overly subjective language should not be used, as well as language that implies intent or culpability (e.g. it is suggested to use ‘deceased’ instead of ‘victim’). Careful proofreading of the report is highly recommended to prevent typographical errors and improve language.

## The elements of the report

The following sections outline the essential elements of the report (‘minimum requirements’) and elements that are optional and may only be useful in certain cases (‘discretionary elements’). Some discretionary elements can be incorporated in a case folder or appendices, rather than in the report text. A summary of the recommendations is provided in Tables 1, 2 and 3.

**Table 1:** Minimum requirements and discretionary elements for the administrative and contextual sections of the report

Element of the report	Minimum requirement	Discretionary elements
Administrative information	Unique case number	Reference numbers from other parties
	Title	Table of contents
	Page number / total number of pages	Short CV of the author
	Lead author’s details, professional address and contact information	Roles and qualifications of people present at the analysis
	Name and contact information of requesting party	
	Date of request	
	Date and location of the analysis	
	Dated signature by the lead author	
	Recipient list of the report	
Contextual information	List of the received contextual sources	Dates of receipt of contextual information
	Author’s summary of contextual information	Number of received photographs
	Question(s) from the requesting party (preferably verbatim)	Summary in chronological order of receipt
		Explicit statement of conditional nature of contextual information

**Table 2:** Minimum requirements and discretionary elements for the materials and methods sections of the report

Element of the report	Minimum requirement	Discretionary elements
Materials	Date and method of receipt of the material Inventory of the received and analyzed skeletal material, including graphical representation or photographs of the received material	Detailed description of received material and/or table of present/absent skeletal elements
	Description of the preservation state of the skeletal material	Detailed description of completeness and preservation
	If applicable, any maceration or other preparation techniques applied to the remains	Date and method of maceration
	If applicable, short list of other received (non-) biological material	Condition before and after maceration
	Dated destination of samples taken during the analysis	Detailed chain of custody, information regarding storage, location, retention periods and disposition of the remains
Methods	Description of which method was applied to which skeletal element	Logical sequence of method descriptions
	For published methods: publication reference	Justification of the chosen method
	For software: name, developer and version	Reference to legal admissibility standards

**Table 3:** Minimum requirements and discretionary elements for the results, discussion, and conclusion sections of the report

Element of the report	Minimum requirement	Discretionary elements
Results	Clearly recognizable in the report	Subdivision in sections for longer reports or those addressing complete skeletal analysis
	Description of all relevant findings, clearly attributed to skeletal material and method, complemented with photographic material if relevant	Tables, diagrams, digital reconstructions, photographs other than those essential for the conclusions
	Description in accordance with the used method	
	Clear distinction between observations and interpretations	
Discussion and Conclusion	Answer to the original question / request, indicating the level of certainty	Detailed discussion of considerations, assumptions and interpretations
	Discussion of relevant limitations and their effect	Consideration of contextual bias Certainty scale glossary
		Executive summary
		Recommendations for further analysis

### Administrative information

#### Minimum requirements

Each case should have a prominently displayed unique case number. The report should have a title, reflective of the subject matter and type of examination, such as “report of forensic anthropological examination of unidentified human skeletal remains” or “forensic anthropological examination of skeletal trauma”. The report pages should be numbered, including the total number of pages (e.g., 1 of 24, 2 of 24, etc.).

The report should have a section with the name, address, and contact information of the lead author or the institution, their title, and their role in the investigation. Furthermore, the name and contact information of the requesting party (e.g. police authority, forensic pathologist) and the date of the request should be included.

The date(s) and location(s) of the analysis should be included. If the analysis was conducted by someone other than the author of the report, their name and qualifications should be stated.

Each report should be signed and dated by at least the lead author. Some jurisdictions have specific requirements, such as witnessed signatures. Failure to comply can render the report inadmissible in court. Lastly, the destination or recipients of the report should be listed.

#### Discretionary elements

Reference numbers from other parties, for instance from police, are preferably included. Longer reports usually benefit from a table of contents. Ideally, the roles and qualifications of those present at the time of examination should be listed in the report.

In some jurisdictions, it is customary to include a short biography of the author, outlining educational background, affiliations, and relevant expertise, including any forensic anthropological certification (e.g., D-ABFA, C-FASE). These biographies are usually included on the front page or early in the report. They should not be exhaustive but highlight case-specific expertise.

### Contextual information

#### Minimum requirements

Each forensic anthropology report should list the contextual sources received (e.g. documents, photographs, witness statements) and contain a summary of relevant context information. If available, the report should outline how the remains were found, as well as context of recovery. When applicable, the description should include a presumptive identity and when an individual was last seen alive. If not described in a separate report, any visits to the scene or excavation site by the forensic anthropologist should be included, preferably under a separate heading. In cases where the forensic anthropology report complements a forensic pathology report, much of the background information may already be included in the medical report. Regardless of this, the forensic anthropology report should remain a standalone document and should include sufficient background information to ensure clarity and interpretability independent of other reports.

This part should also include the aim of the analysis and any specific questions or issues to be addressed (preferably under a separate heading and as formulated in the formal request).

#### Discretionary elements

A more detailed description of contextual information is often recommended. If described or named, scene photographs should be numbered.

Separating information which was received before and after the analysis can be helpful to justify the methodological approach and interpretation at a later date. Because contextual information often changes, it is recommended to summarize it in the order it was received. This may help address later concerns about contextual bias, in case any arise. It may also be helpful to explicitly state that the summary is based on the available information and may change if new information becomes available.

Direct quotes (using quotation marks or italics) from source material can enhance data integrity but should be clearly referenced (e.g., in footnotes).

### Examined items

#### Minimum requirements

Each report should describe the skeletal items under study, especially since the report may eventually be the only traceable record of the skeletal remains and their examination and may concern only a subset of the recovered material. Also, forensic anthropology examinations are often part of prolonged or reopened investigations. Anthropological reports may therefore be crucial when considering re-examination or exhumation. The inventory may also help clarify the methodological choices made.

The report should state when and how the material for examination was received. A detailed chain of custody, as well as information regarding storage, location, retention periods and disposition of the remains do not need to be included in the report and may instead be maintained within the case folder. Such information should be made available upon request.

At a minimum, the body of the report should include a dedicated inventory section, describing the received items and which of these were analyzed. The format of the inventory may vary and can be presented as text, tables, photographs or diagrams. The textual description may be brief but should be complemented by a more detailed table (e.g., in an appendix) and/or photographic or diagrammatic documentation. The preservation status of the remains (e.g., skeletonized, burned) should be described, together with any non-biological items submitted with or recovered alongside the remains (e.g., personal effects). Where skeletal preparation techniques (e.g., maceration) have been performed prior to analysis, or if samples are taken during the analysis for other examinations, this should be clearly specified.

#### Discretionary elements

A more detailed description of the examined material can include completeness and a detailed description of preservation status which may further strengthen and clarify the analytical approach and limitations, without having to request case folder materials. More detailed tables and photographs of the analyzed material can be included in an appendix.

When macerating, it may be helpful to document/describe the condition of the remains before and after the procedure. A brief description of the method used is recommended followed by the corresponding reference if the method is published. Alternatively, a maceration protocol can be provided in an appendix.

### Methodology

#### Minimum requirements

The validity of most forensic anthropological methods is case-dependent. Methods are, for instance, population-specific, limited to certain skeletal elements, or only applicable when the material meets certain preservation criteria. Consequently, choice of methodology is a core aspect of forensic anthropological analysis.

At a minimum, the report should clearly describe the method(s) and the skeletal element(s) to which it was applied. The description should be sufficiently detailed to enable reproducibility if required.

Typically, methods are derived from published sources. In such situations, the references that demonstrate the validity of the method should be provided. Any deviations from the original method should be described. The use of digital decision support systems (e.g., Osteomics, Fordisc) should be documented, inclusive of version number.

Sometimes a method is specifically developed for the case. In such a case, the methodology section should discuss the lack of appropriate published methods, provide a detailed description of the developed method, and justify its reliability and accuracy.

Supplementary examinations involving methods not typically included within the standard forensic anthropological approach (e.g., histology or imaging) may be reported either in this section or under a separate dedicated heading.

When only well-known methods are applied to one or a few skeletal elements, a detailed methodology section may not be necessary. The method(s) can then be referenced succinctly in the results, with citations provided in a reference list or footnote.

#### Discretionary elements

The order in which methods are described is at the author’s discretion, but maintaining a logical sequence reflective of the analysis conducted is recommended. For instance, almost all stature estimation formulae are sex-, age-, and population specific, therefore stature is reported after the other elements of the biological profile are assessed and explained in the report. When multiple analyses address the same issue (e.g. trauma), macroscopic examination is generally first, followed by radiology or microscopy. Using subheadings within the analysis section can improve readability of the report.

To accommodate the diverse readership of the report, it may be helpful to justify the chosen methodology by explaining factors such as population specificity, prior application in similar cases, or demonstrated scientific rigor. This helps the reader to understand the methodological approach but can also assist the author when answering questions at a later date. If applicable, a legal admissibility standard is best explicitly cited, with an explanation of why the methodology conforms.

### Results

#### Minimum requirements

The results of the analysis (observations) should be described clearly, objectively, and in accordance with the method used. For example, if a method provides a numerical result with a confidence interval, both should be reported. If a method produces a score that can be used to infer a category (e.g., sex), both the score and the estimated sex should be included. All reported observations should be attributable to the examined items and the methods described.

All relevant observations should be reported, even when they are negative or inconclusive. For example, if trauma is assessed but not observed, this should be explicitly stated. Similarly, when multiple methods are applied to the same analytical aspect (e.g., sex or age estimation), the observations from each analysis should be reported in cases where no single method is demonstrably more accurate. Where a method with high accuracy is applicable (e.g., pelvis for sex estimation), results from other methods may be omitted from the report and retained within the case folder.

The description of observations should allow another practitioner to make their own independent interpretations. This requires a separation of observations and opinions wherever possible. This is easy for metric results, such as measurements or volumes, but many forensic anthropological observations are inherently descriptive. For example, description of morphological sexually dimorphic traits, interpretations of skeletal trauma, and diagnoses of disease all require interpretative judgment. In such cases, observations should be sufficiently detailed to support peer review. For example, trauma descriptions should focus on specific fracture characteristics (e.g., orientation and location of fractures), including biomechanical response (plastic deformation), rather than directly inferring biomechanical causes (e.g., compression or tension). Similarly, observations regarding skeletal pathologies should be described as relevant positive and negative observations that inform differential diagnosis, rather than directly concluding a diagnosis.

Photographic documentation should be included where it is relevant to the investigation. For example, photographs of features contributing to the biological profile in cases of unidentified remains or images of perimortem trauma should be provided, whereas photographs of elements not central to the investigation (e.g., documentation of postmortem defects) are not required. Results presented in this way facilitate any later review process and support transparency. When including photographs, images should be of high-quality, with a uniform background, a scale, and a clear label or contextualization of the elements. Pertinent features should be preferably marked.

#### Discretionary elements

Where appropriate, the observations can be organized in sections. Depending on the examination, typical subheadings may include ‘medico-legal significance’; ‘post-mortem interval estimation’; ‘minimum number of individuals’; ‘taphonomy’ ‘biological profile (i.e., population affinity, sex, age, stature)’; ‘skeletal trauma’; ‘identification features (e.g. dental status, pathology, anatomical variants, antemortem trauma)’; and ‘ancillary investigations’ (e.g., radiology, isotopes, histological analysis, DNA testing).

If multiple analyses address the same element of the examination (for instance, age estimation), it is helpful to outline how they are combined.

Results can also be grouped based on the methods of analysis. For instance, a distinction between macroscopic, radiological, and histological observations can be appropriate. Alternatively, the histological and radiological observations can be included under a separate heading dedicated to supplementary analysis.

Tables, figures, and diagrams are very effective in communicating complex data and are therefore encouraged. When used correctly, they facilitate easy navigation and can demonstrate connections in the data that are difficult to explain textually. Similarly, judicious use of photographs can simplify or circumvent complex descriptions. When reporting on crania, photographs of all six aspects are encouraged. Other representations of skeletal items (e.g., 3D reconstruction, photogrammetry) can also be included.

All tables, figures, and photographs should be numbered and referenced in the text. They should be understandable stand-alone, with a descriptive title and explanatory caption.

Examination logs, laboratory notes, worksheets, and software outputs do not need to be part of the report but should be retained and made available upon request. Naturally, the information in the case folder should concur with the information in the report.

### Discussion and conclusion

#### Minimum requirements

The extent of this section will depend on the nature and complexity of the case. Some cases may not require a discussion; a short concluding paragraph suffices. At a minimum, such a conclusion should clearly address the original question(s) in the request (listed in the contextual section of the report). The inferential basis of the opinion should be provided in a language that reflects the level of certainty of the author of the report. When results are inconclusive or limited, the reason for this should be explained.

The report should mention relevant limitations that impacted the examination. This should also include their effect on the validity of the examination. Determining whether a limitation is relevant is inherently subjective, meaning that practitioner’s opinions may differ about what to include, even when they agree on the analysis and observations. As a rule of thumb, limitations should be discussed when they could reasonably influence how the recipients of the report weigh the examination’s conclusions. Discussing limitations can be challenging, since limiting factors often coexist and interact. Examples of methodological limitations include possible deviations from published reliability and accuracy levels, especially when using reference data not derived from the same population as the decedent. Other case-specific limitations can complicate matters further, such as variability in preservation or incomplete contextual information.

The discussion and conclusion should be strictly based on the context, items, analysis, and observations described in the report. New observations or data should not be introduced. Furthermore, the discussion should not address aspects that are beyond the expertise of the author or the scope of the examination. For example, conclusions on the cause of death are typically the prerogative of a forensic pathologist.

Often, the conclusion is the most read part of the report. Care should therefore be taken to minimize the risk of misinterpretation when it is read on its own.

#### Discretionary elements

In complex reports, it is recommended to include a detailed discussion of the considerations, assumptions, and interpretations that underpin the opinions. Special consideration can be given to the effect of context information, whether pertinent information was missing, or whether domain-irrelevant information may have affected the analysis. All commentary should be balanced: not every potential source of uncertainty needs to be discussed, but significant limitations should be addressed.

Where a certainty scale is applied, a glossary defining its levels can be included.

The discussion may recommend further analysis, such as histology, isotope testing, or DNA analysis, or referral of the material to a different specialist.

The ISO Standard 21,043 ‘Forensic Science’ specifically addresses interpretation [13] and may be of relevance for the reporting of forensic anthropological examinations. The standard outlines the logically correct method to evaluate forensic science observations, based on Bayes’ theorem. This approach is also known as ‘the likelihood-ratio framework’ for its use of likelihood ratios, which expresses a practitioner’s assessment of the strength of the presented evidence. Various publications have demonstrated its usefulness in forensic anthropology, such as biological profiling, Disaster Victim Identification (DVI), and trauma analysis [32–34].

Depending on the scope and audience of the report, an executive summary or tabulated overview of the main conclusions may be helpful. This particularly applies to very long reports.

### Archiving

#### Minimum requirements

All reports should be archived securely, in an access-controlled digital or physical environment, together with all source information (e.g., case folder) that is essential to verify the report’s conclusions independently. The report should remain easily accessible and identifiable. The minimal duration of storage will depend on local legal requirements, but the aim should be to store these materials indefinitely. Access to the report will also be dictated by local laws and regulations. In the absence of such rules, the report should remain confidential, and permission should be sought before it is made available to others than the allowed parties.

A record should be kept when reports are supplemented or amended. Supplementary or amended reports should be clearly recognizable and identify what has been changed. A record should also be kept if a report is withdrawn, explaining the reason for the retraction.

#### Discretionary elements

Where electronic storage of the report is mandatory, retention of a physical copy is discretionary. This also holds for any supportive, but non-essential material related to the case. Storage of such materials is highly recommended and should be outlined in standard operating procedures. This can help to address issues arising at a later date when not mentioned in the initial report. In addition, new analytical techniques may become available, prompting a re-assessment of the case.

## The structure and the extent of the report

Notwithstanding the above recommendations, a practitioner can decide to adopt a different structure of their report. For example, a separation of contextual information, examined items, analytical methods, observations, and opinions does not necessarily mean that these should always be provided under separate headings. An alternative approach in which sections are dedicated to specific forensic issues (e.g., age at death or sex estimation) can also be used. These sections would then outline the analysis, observations, and interpretations relevant to that issue. The format of the report may also vary when it is limited to a specific question (e.g., trauma analysis), as the content should be guided by the questions posed in each case and should not include elements that fall outside the scope of the investigation.

Deviations from the above recommended structure can also stem from the nature of the request. Straightforward analyses, with observations that require limited interpretation, can be appropriately and sufficiently communicated by a few short statements. Such short reports may, for instance, be useful when remains are non-human or of no medico-legal significance. Short reports have the added benefit that they can be produced quickly, giving timely guidance to police or other entities. Even short reports should, however, be clearly structured, outlining circumstances, materials, observations, and should function as standalone documents containing the author’s definitive opinion. For a human/non-human request, the circumstances, materials, observations can be combined in a statement such as the following:

*Based on the visual analysis of the [insert examined item]*,* including the shape of the joint surface*,* developmental stage of the remains and the size of the bone*,* the skeletal element was determined to be of non-human origin*.

Reference can also be made to a standard human anatomy atlas.

Short reporting should not result in ambiguity, and laboratory records should be sufficiently detailed so that a more comprehensive report can be produced if required. Short reports may lend themselves to verbal communication. It is, however, essential to keep a written record of what was said, to whom, at what date and time. As for other reports, all supporting material, including photographs, should be retained.

## Conclusions

Consistent, transparent, and well-reasoned reporting is essential not only for effective communication with medico-legal stakeholders, but also for safeguarding the scientific credibility and legal robustness of forensic anthropological evidence. These recommendations aim to balance standardization with the flexibility required by diverse legal frameworks, institutional settings, and case complexities. The implementation of these recommendations will enhance reporting quality, facilitate peer review, and ultimately strengthen the role of forensic anthropology within the justice system.

## Data Availability

No datasets were generated or analysed during the current study.
